# Point-of-care Ultrasound to Identify Distal Ulnar Artery Thrombosis: Case of Hypothenar Hammer Syndrome

**DOI:** 10.5811/westjem.2015.4.25888

**Published:** 2015-07-02

**Authors:** Jonathan Ken, Darshan Khangura, Sean P. Stickles

**Affiliations:** * University of Missouri, Department of Emergency Medicine, Columbia, Missouri; † University of Missouri, Department of Internal Medicine, Columbia, Missouri

## Abstract

Hypothenar hammer syndrome (HHS) is a rare condition of distal ulnar artery injury and thrombosis secondary to repetitive blunt trauma to the hypothenar area. We present a case of HHS for which point-of-care ultrasound (POCUS) was used as the initial means of imaging, prompting management and disposition without further imaging studies ordered in the emergency department (ED). This case demonstrates the utility of POCUS to aid the Emergency Physician in the diagnosis and management of patients with extremity vascular issues in the ED, and details a rarely seen clinical entity in the ED.

## INTRODUCTION

First described by Von Rosen in 1934 and coined by Conn et al. in 1970, hypothenar hammer syndrome (HHS) is a rare condition of distal ulnar artery injury and thrombosis secondary to repetitive blunt trauma to the hypothenar area.^1^–^2^ The condition typically occurs in middle-age men with occupations that expose the hand to repeated trauma.^3^ Affected patients present with signs and symptoms of hypoperfusion to digits perfused by ulnar artery branches (digits 3–5). We present a case of a patient with distal ulnar artery thrombus diagnosed by point-of-care ultrasound (POCUS) in the emergency department (ED), ultimately determined to be most consistent with a case of HHS. Treatment and disposition were determined based on POCUS imaging alone, without additional studies ordered through the ED.

## CASE REPORT

A 27 year-old man with a history of endocarditis and mechanical aortic valve replacement presented to the ED with a chief complaint of numbness and coldness to his right 4^th^ and 5^th^ digits for 2 weeks. The patient reported his symptoms started by noting a painful area of swelling to his right hypothenar area, followed over the course of the next week by development of the numb and cold sensations to his 4^th^ and 5^th^ digits. The swelling had since resolved, but the hypothenar area was still painful. The patient denied trauma to the area or a history of similar symptoms in the past. The patient reported inconsistent compliance with taking warfarin for his mechanical valve. The patient’s social history was significant for smoking tobacco and working as a mechanic; he denied current recreational drug use. The patient denied significant family medical history. On exam, the patient’s 4^th^ and 5^th^ digits were pale, cool to the touch, and exhibited prolonged capillary refill. The patient also reported decreased sensation to the affected digits. A 2×2mm eschar area was noted to the distal 4^th^ digit on the volar surface. No other digital lesions, petechiae, or nodules were noted. The hypothenar area on the affected hand was tender to touch, although no swelling was appreciated. A loud S2 click was audible on cardiac auscultation; no additional murmurs were appreciated. Vital signs were notable for a temperature of 38.3°C and tachycardia to 110 beats per minute. The remainder of the exam was unremarkable.

**Figure 1:**
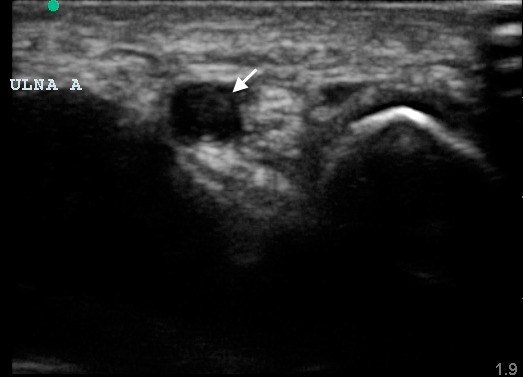
Ultrasound image of the distal ulnar artery in transverse plane noting echogenic thrombus (arrow) within the vessel lumen.

**Figure 2:**
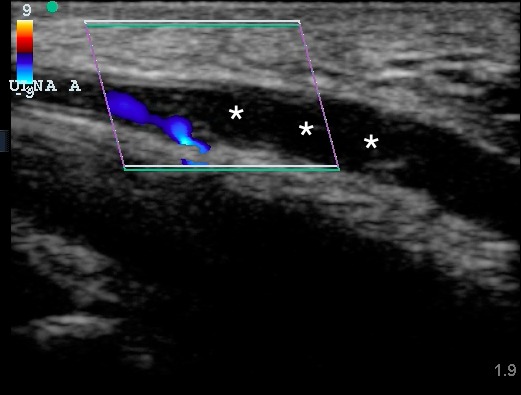
Ultrasound image of the distal ulnar artery in longitudinal plane noting reduced flow proximal to the thrombus with absent flow distally and echogenic thrombus (asterisks) within the lumen.

Blood work sent was remarkable for a mild leukocytosis of 12.9 ×10^9^/L and a subtherapeutic international normalized ratio (INR) of 0.9. A POCUS of the right distal ulnar artery was performed using a high frequency linear transducer and noted an area of mixed echogenicity within the lumen of the ulnar artery extending from the ulnar-carpal junction into the hypothenar area, suspicious for occlusive thrombus (Figure 1). The vessel was noted to retain compressibility proximal to the mixed echogenic focus, but it was incompressible over the area of the suspected thrombus. Color Doppler confirmed a lack of flow at the area of suspected thrombosis (Figure 2, Video). Based on the clinical and POCUS findings, Hand Surgery was consulted and the admitting Medicine team was notified. Both services agreed with the diagnostic finding of ulnar artery thrombosis and appreciated the need for further evaluation into its etiology on the inpatient service. In the ED, the patient was started on a heparin infusion and antibiotics until recurrent endocarditis could be ruled out. The following day, the patient underwent a computed tomography angiogram (CTA) of the right upper extremity which confirmed localized thrombus at the ulnar artery at the level of the carpals with diminished flow to the 4^th^ and 5^th^ digits. A transesophageal echocardiogram was also performed which noted no valvular vegetative lesions. Blood cultures remained negative for growth after 48 hours. Following the patient’s inpatient evaluation and supported by the patient’s occupational history of being a mechanic, the clinical scenario was determined to be consistent with HHS. Interventional Radiology was consulted who recommended no immediate intervention as the patient exhibited no evidence of progressive or severe ischemia. The patient resumed his home dose of warfarin, was started on aspirin and nifedipine, was advised to stop smoking, and was discharged for outpatient follow up. At his first appointment 2 weeks after discharge, the patient reported no worsening of his symptoms, and was recommended to continue medical therapy and follow up in 4–6 weeks. The patient did not keep his next appointment.

## DISCUSSION

HHS is a rare syndrome of digital ischemia caused by damage to and thrombosis of the distal ulnar artery as it courses through Guyon’s canal and around the hook of the hamate bone.^3^–^4^ The damage to the artery typically results from recurrent blunt trauma to the hypothenar area of the hand. HHS predominantly occurs on the dominant hand of middle-age men.^5^ Certain occupations pose a particular risk to the development of HHS, including individuals working as auto mechanics, blacksmiths, metal workers, and butchers.^5^ It has also been noted in athletes that expose the hand to repeated trauma (e.g. hockey, softball, football, badminton).^6^–^8^ Affected patients typically complain of symptoms of pain, numbness, tingling, cold intolerance, and weakness of their 3^rd^-5^th^ digits.^3^,^9^ Signs of HHS may include digital pallor, cyanosis or mottling, a palpable hypothenar mass (representing thrombus), atrophy of distal finger pads, and fingertip findings, such as splinter hemorrhages, ulcerations, and gangrene.^10^ The male predominance, occupational exposure, and asymmetric distribution help to distinguish it from other vascular disorders (i.e. Raynaud’s phenomena, thoracic outlet obstruction, arterial emboli).^3^ Management for mild cases is largely supportive including lifestyle modification (i.e. smoking cessation, using gloves during work), antiplatelet and anticoagulant medications, and calcium channel blockers to reduce vasoconstriction.^3^,^10^ More severe cases or those that fail conservative management may require operative repair including vessel resection with ligation, grafting, or thrombolysis.^3^

Allen’s test can be useful to assess for ulnar artery patency when evaluating for HHS, although has been reported to be normal in up to 14% of cases.^3^ Arteriography is considered the traditional “gold standard” for diagnosis, often noting a characteristic “corkscrew” appearance of the affected portion of the artery as it courses along the hook of the hamate, and may be useful for operative planning.^3^ However, its invasiveness and high cost make it impractical to order as the initial test for evaluation. Therefore, CTA and Ultrasound are the primary imaging modalities used for evaluation of HHS. While CTA provides excellent imaging of the artery and can be useful to assess digital perfusion, drawbacks include higher cost and patient exposure to ionizing radiation. POCUS has been proven as an effective means of evaluation of various vascular pathologies including thrombosis, aneurysm, pseudoaneurysm, and dissection.^11^–^14^ While HHS has been previously described in Emergency Medicine literature, none of these reports highlight the potential usefulness of POCUS for its evaluation.^15^–^16^ In the described case, we used POCUS to identify the area of transition of the ulnar artery from normal to occluded vessel, occupied by a visualized thrombus. Non-compressibility of the vessel sealed the diagnosis of thrombotic occlusion. While the evaluation into the possible etiology of the occlusion would warrant admission and further workup (given the patient’s risk factors of previous endocarditis, mitral valve replacement, subtherapeutic INR, smoking and occupational histories), the results of the POCUS study allowed for the expedition of medical management and disposition. This case highlights the value in utilizing POCUS as the initial imaging study of potential extremity vascular issues.

## CONCLUSION

HHS is a condition of distal ulnar artery injury and thrombosis secondary to repetitive trauma to the hypothenar area. In this case report, we demonstrate the convenience and utility of using POCUS as the initial diagnostic test for distal extremity arterial thrombosis. Based on our POCUS findings, we were able initiate medical management and collaborate with the consulting and admitting teams quickly. While the patient had multiple risk factors for other etiologies of ulnar artery occlusion, (e.g. emboli from recurrent endocarditis and mechanical valve replacement, smoking history) an exhaustive work-up ruled these out and the etiology was ultimately determined to be most consistent with HHS. Although HHS is a rare entity, emergency physicians (EPs) should be vigilant of the condition during the evaluation of signs and symptoms of digital ischemia, particularly when they are in the ulnar artery distribution. Additionally, EPs should incorporate POCUS into their evaluation of these complaints as this affords a rapid and effective means of diagnosis and can hasten treatment.

## Figures and Tables

**Video:** Ultrasound clip of the distal ulnar artery in longitudinal plane noting reduced pulsatile flow proximal to the thrombus with absent flow distally and echogenic thrombus within the lumen.
